# Langzeitverläufe bei Schizophrenien: Eine Übersicht zu aktuellen Befunden und Entwicklungen

**DOI:** 10.1007/s00115-024-01790-7

**Published:** 2024-12-19

**Authors:** Igor Nenadić, Irina Falkenberg, Stephanie Mehl, Tilo Kircher

**Affiliations:** Klinik für Psychiatrie und Psychotherapie, Philipps Universität Marburg & Universitätsklinikum Gießen und Marburg (UKGM), Rudolf-Bultmann-Straße 8, 35039 Marburg, Deutschland

**Keywords:** Bildgebung, Kognition, Neuropsychologie, Pharmakotherapie, Psychotherapie, Imaging, Cognition, Neuropsychology, Pharmacotherapy, Psychotherapy

## Abstract

Entgegen der klassischen Konzeptualisierung der Schizophrenie als eine progressive Erkrankung mit hoher Chronifizierung zeigen aktuelle Langzeitstudien einen höheren Anteil an Remissionen, möglicherweise auch einen höheren Anteil an Recovery als bisher angenommen. Die Heterogenität der klinischen Verläufe spiegelt sich auch in kognitiven und biologischen (z. B. bildgebenden) Variablen wider, in welchen viele Betroffene Remissionen zeigen. Frühe Interventionen in den ersten Wochen und Monaten nach Erkrankungsbeginn sind dabei nicht nur für die unmittelbare Remission, sondern möglicherweise auch für den Langzeitverlauf entscheidend. Die Behandlung und Reduktion somatischer Begleiterkrankungen ist neben einer differenzierten Kerntherapie dabei ein vielversprechender Ansatz, Verläufe auch nach Jahren der Erkrankung positiv zu beeinflussen. Die Identifikation weiterer Prädiktoren, z. B. anhand biologischer Parameter, kann dabei zusammen mit Ansätzen des maschinellen Lernens zur Optimierung einer individualisierten Kerntherapie beitragen.

## Abstract (en)

Despite classical conceptions of schizophrenia as a progressive illness with a high chronification, current long-term follow-up studies show a higher proportion of remission, possibly also a higher proportion of recovery than previously assumed. The heterogeneity of clinical courses is also reflected in different trajectories of cognitive and biological (e.g., imaging) variables, in which many of those affected show remission. Early clinical intervention during the first weeks and months following the onset of psychosis are decisive not only for early remission but also possibly for the long-term outcome. The treatment and reduction of somatic comorbidities are promising approaches in addition to a differentiated core treatment to positively influence the course of the illness even years after the disease. The identification of additional predictors, e.g., based on biological parameters, can together with machine learning approaches contribute to optimization of an individualized core treatment.

## Hintergrund

Die Dementia praecox als klinisches Vorläuferkonstrukt der Schizophrenie(n) hat wesentlich den ungünstigen Krankheitsprozess als zentral in der Abgrenzung zu affektiven Störungen wie auch anderen „Psychosen“ bzw. schweren psychischen Störungen gesetzt, welcher durch schwere funktionelle bzw. kognitive Einschränkungen definiert worden war [40]. Nicht erst seit den früheren Verlaufsstudien von E. Bleuler, wie auch beispielsweise der ABC-Studie von Häfner und Kollegen [37], hat sich gezeigt, dass die Entwicklung von Symptomen und Beeinträchtigungen, vom Prodrom über Erstmanifestation und Verlauf der nachfolgenden Jahre, von einer erheblichen Heterogenität gekennzeichnet ist [15, 41, 61].

Die aktuelle Forschung zum Verlauf schizophrener Erkrankungen ergänzt die Optimierung deskriptiver Verlaufsuntersuchungen: einerseits um zuverlässigere prospektive Prädiktoren der Trajektorien klinischer und neurobiologischer Parameter, andererseits mit der Untersuchung therapeutischer Interventionen auf ihre Effekte zur tatsächlichen Modifikation des Krankheitsverlaufs und nicht nur (temporärer) Symptomreduktion [59]. Hierzu gehören auch Ansätze zur Optimierung von Komorbiditäten, welche eine bedeutende Rolle in der klinischen Versorgung chronisch Kranker einnehmen, sowie auch die Frage von Recovery.

Die vorliegende nichtsystematische Übersichtsarbeit soll aktuelle Studien zur Frage des Langzeitverlaufs der Schizophrenie über Jahrzehnte hinweg unter diesen Gesichtspunkten zusammenfassen. Hierbei soll auch deutlich werden, ob und wie aktuelle Ansätze einer Komplexbehandlung der Schizophrenie zur Verbesserung der Langzeitverläufe beitragen. Schon frühere Studien, etwa von G. Huber und Mitarbeitenden, wiesen auf die hohe Heterogenität klinischer Verläufe bei Schizophrenie hin, was einen wichtigen Ansatzpunkt für die Optimierung von Outcomes über die Lebensspanne darstellt (Übersichten in [34, 45]).

## Psychosoziales Funktionsniveau und Psychopathologie

Zum Verlauf von Symptomen und Funktionsniveaus liegen insbesondere mit der AESOP-10- und OPUS-Studie neue Daten vor, welche einige frühere Befunde präzisieren und teilweise revidieren. AESOP-10 untersuchte über 550 Personen von der Erstmanifestation einer psychotischen Episode (Schizophrenie und andere nichtorganische Störungen mit psychotischen Symptomen) in drei Städten Englands über einen 10-Jahres-Zeitraum. Während in den 10 Jahren nach Erstmanifestation einer psychotischen Episode ein erheblicher Anteil von ca. 77 % aller Betroffenen stabile Remissionen (i. S. eines Fehlens psychotischer Symptome) über zumindest einen längeren Zeitraum zeigte, war doch ein ähnlich hoher Anteil von 72 % im psychosozialen Funktionsniveau so eingeschränkt, dass die Betroffenen über den Großteil des Follow-up-Intervalls keiner geregelten Berufstätigkeit nachgehen konnten [64]. In einer nachfolgenden Teilanalyse der Kohorte konnten vier prototypische Trajektorien abgegrenzt werden, wobei 58,5 % einen remittierend verbessernden Verlauf nach Erstmanifestation und 30,6 % einen persistierenden Verlauf zeigten (weitere 5,6 % eine späte Verschlechterung und 5,4 % eine späte Verbesserung; [63]). Hierbei ist zu erwähnen, dass nicht alle der in AESOP-10 eingeschlossenen Personen initial (oder im Verlauf) eine Schizophreniediagnose erhalten hatten, sondern auch Betroffene mit einer psychotischen Episode im Rahmen etwa einer affektiven Störung eingeschlossen worden waren. Wenngleich bei Personen mit psychotischer Symptomatik im Rahmen einer affektiven Störung die Trajektorien deutlich günstiger sind als bei Schizophrenien [13, 30, 42], sind insgesamt doch Remissionsraten bei Schizophrenie höher als in früheren Studien geschätzt und es zeigen sich auch bei einem erheblichen Anteil dieser Personen längere Phasen geringer Symptombelastung bzw. Funktionseinschränkungen, sodass nach ca. 10 Jahren knapp die Hälfte der ursprünglich eingeschlossenen Erkrankten eine symptomatische (46,2 %) Recovery bzw. eine Recovery des Funktionsniveaus (40,9 %) erreicht [13].

### Infobox Remission und Recovery

Remission und Recovery sind zentrale Konzepte in der Behandlung von Schizophrenie, die jedoch unterschiedliche Bedeutungen haben.

Die Konsensuskriterien der Remission der Schizophrenia Working Group (RSWG; [2]) definieren Remission als ein Niveau der Kernsymptome der Schizophrenie, welches das Verhalten einer Person nicht beeinträchtigt und unterhalb der gemäß Diagnostic and Statistical Manual of Mental Disorders (DSM) IV für die Diagnose einer Schizophrenie erforderlichen Schwelle liegt. Sie bestehen aus zwei Elementen:

1. Ein symptombezogenes Kriterium, bestehend aus sieben diagnostisch relevanten Items des DSM-IV.
  Diese Items wurden mit drei verschiedenen Bewertungsskalen abgeglichen: der Positive and Negative Syndrome Scale (PANSS), der Scale for the Assessment of Negative Symptoms and Positive Symptoms (SANS/SAPS) sowie der Brief Psychiatric Rating Scale (BPRS). Sie entsprechen folgenden acht Items der PANSS, die alle mit einer Symptomschwere von ≤ 3 Punkten („mild“ oder besser) bewertet sein müssen:
  - Wahnideen,
  - ungewöhnliche Denkinhalte,
  - Halluzinationen,
  - formale Denkstörungen,
  - Manierismen und unnatürliche Körperhaltung,
  - Affektverflachung,
  - soziale Passivität und Apathie,
  - mangelnde Spontaneität und Flüssigkeit der Sprache.

Das symptombezogene Kriterium kann auch mittels SANS/SAPS bewertet werden (Schweregrad ≤ 2 Punkte). Die BPRS (Schweregrad ≤ 3 Punkte) enthält jedoch keine adäquate Repräsentation der Negativsymptomatik und ist daher allein nicht ausreichend für die Bewertung der Remission. Die beiden Negativsymptome, die in der BPRS fehlen („soziale Passivität und Apathie“ und „mangelnde Spontaneität und Flüssigkeit der Sprache“), müssen daher zusätzlich mittels PANSS oder SANS bewertet werden.

2. Ein zeitliches Kriterium, das verlangt, dass die symptombezogenen Kriterien für mindestens 6 Monate erfüllt sind.

Eine *Remission* kann symptomatisch sein, was bedeutet, dass z. B. die psychotischen Symptome reduziert sind, aber nicht notwendigerweise auch die psychosoziale Funktionsfähigkeit oder Lebensqualität des Patienten verbessert ist.

Daher beschreibt *Recovery* neben der symptomatischen Remission auch die funktionale und psychosoziale Erholung [57], was bedeutet, dass Patienten in der Lage sind, ein erfülltes und selbstbestimmtes Leben zu führen. Auch weitere Aspekte wie das Streben nach Sinn und Erfüllung, Hoffnung, Gleichberechtigung und Selbstbestimmung sind Teile von Recovery als einer stärker individualisierten und patientenzentrierten Perspektive, wobei validierte Kriterien für Recovery bislang fehlen.

Als ein auch für die klinische Praxis relevanter und wichtiger Prädiktor des Langzeitverlaufs konnte in AESOP-10 die Symptomremission 12 Wochen nach initialer psychotischer Episode identifiziert werden; dies bezog sich auf die Verbesserung der Symptomlast wie auch des psychosozialen Leistungsniveaus [13]. Weitere Analysen zeigten, dass neurologische „soft signs“ bei Ersterkrankung überdurchschnittlich häufig bei Personen mit einem schlechten klinischen Outcome zu finden waren [8, 21], ähnlich dazu war bei kognitiven Defiziten das Risiko eines nachfolgenden Verlaufs mit Behandlungsresistenz erhöht [50].

Aktuelle Prädiktionsmodelle (unter Einbeziehung von AESOP) zeigen, dass bei Erstmanifestation einer psychotischen Episode bereits einige klinische Parameter eine recht gute statistische Schätzung des Auftretens späterer Behandlungsresistenz (und damit schlechterer Verläufe) ermöglichen, wobei zu diesen Merkmalen junges Alter der Erkrankten, Dauer der unbehandelten psychotischen Episode, Drogenkomorbidität, Alkoholkonsum, prämorbides Funktionsniveau, Diagnose einer Schizophrenie bzw. schizoaffektiven Störung bei Erstmanifestation sowie Familienanamnese einer psychotischen Störung zählen [19].

Mit der dänischen OPUS-Studie liegen mittlerweile nun auch klinische, kognitive und andere Daten über einen Zeitraum von bis zu 20 Jahren vor, welche Aussagen über Effekte der frühen klinischen Intervention auf den Verlauf erlauben. Über den Nachweis prototypischer Verlaufstrajektorien hinaus konnte dabei auch gezeigt werden, dass sich unterschiedliche Trajektorien auf der Ebene von Positiv- vs. Negativsymptomen abgrenzen lassen [80]: Bei Positivsymptomen zeigten die meisten Ersterkrankten entweder frühe kontinuierliche Verbesserungen (50,9 %), stabile Verbesserungen (18 %) oder aber eine temporär auftretende Symptomatik (10,2 % intermittierend und 11,9 % Rückfälle mit mäßiger Symptomlast), jedoch nur 9,1 % tatsächlich über längere Zeiträume persistierende Positivsymptome; bei den Negativsymptomen dominierten zwei typische Verlaufsbilder mit einer Remission bei 51 % und Persistenz bei 49 %. Analog dazu findet sich bei akustischen Halluzinationen wie auch psychosozialem und kognitivem Funktionsniveau ein erheblicher Anteil an Personen mit einer dauerhaften bzw. über lange Zeiträume erhaltenen bzw. sich bessernden Funktion [49, 79]. Unklar bleibt hingegen, ob Frühinterventionsprogramme einen langanhaltenden Effekt bei Schizophrenie zeigen können, zumal in der OPUS-Studie trotz kurzfristiger Vorteile bei Teilnehmenden einer zweijährigen gezielten Frühintervention dann nach 20 Jahren kein signifikanter Unterschied zu nicht in diesem Programm behandelten Betroffenen nachzuweisen war [39]. Hier ist möglicherweise der Zeitverzug bis zum Beginn einer gezielten Intervention maßgeblich [1]. Ein weiterer ganz wesentlicher Aspekt betrifft die Reduktion bzw. den Verzicht auf Cannabiskonsum nach Ersterkrankung, was in zwei großen Ersterkrankungsstudien zu einem signifikanten Vorteil im 5‑ bzw. 10-Jahres-Follow-up nachweislich beitrug [9, 77].

Einschränkend ist zu betrachten, dass mittlerweile die meisten dieser Langzeitstudien Menschen mit einer ersten psychotischen Erkrankung bzw. Episode einschließen und damit nicht nur Personen mit einer Schizophreniediagnose. Letztere zeigt zwar auch in anderen epidemiologischen Verlaufsstudien eine deutlich geringere Rate an stabiler Recovery bzw. Genesung im Vergleich zu anderen psychotischen Störungen (z. B. auch im Suffolk County Mental Health Project [82]), jedoch zeigen die neueren Studien zu Langzeitverlaufstrajektorien die Möglichkeit einer dauerhaften Besserung für einen nicht unerheblichen Teil der Betroffenen und ergänzen damit frühere Langzeitstudien [6], welche zahlreiche der genannten Prädiktoren schon diskutiert hatten.

Gemäß der Empfehlung von Menzez und Kollegen [57] legen Studien zum Langzeitoutcome bei Erkrankungen aus dem Schizophreniespektrum in den letzten Jahren zunehmend eine multidimensionale Definition von Recovery zugrunde, welche neben der klinischen Remission auch funktionelle Aspekte beinhaltet. Jääskeläinen et al. [48] empfehlen darüber hinaus auch ein Zeitkriterium der anhaltenden Verbesserung klinischer, funktioneller oder beider Aspekte von mindestens 2 Jahren anzulegen. Unter Verwendung dieser Kriterien fanden sie in einem systematischen Review mit Metaanalyse eine im Vergleich zu früheren Untersuchungen niedrigere Genesungsrate von 13,5 % ohne statistisch signifikante Unterschiede in Bezug auf Geschlecht, Zeitpunkt der Datenerhebung, Dauer der Nachbeobachtungsperiode, Vorliegen einer Erstmanifestation und Qualität der zugrunde liegenden Studien. Die mediane jährliche Genesungsrate lag bei 1,4 %, was vereinfacht ausgedrückt bedeutet, dass von 100 Personen mit Schizophrenie 1 bis 2 Personen pro Jahr die genesungsbezogenen Kriterien erfüllen würden, und etwa 14 % würden voraussichtlich innerhalb von 10 Jahren genesen.

Übereinstimmend mit früheren systematischen Übersichtsarbeiten (z. B. [41]) fanden die Autoren keine Hinweise auf einen Anstieg der Genesungsraten über die letzten Jahrzehnte bzw. sogar numerisch sogar eine Abnahme über den Verlauf der letzten 50 Jahre. Angesichts deutlich verbesserter Therapie- und Versorgungsmöglichkeiten für Menschen mit Schizophrenie erscheint dieses Ergebnis zunächst ernüchternd, ist aber möglicherweise dadurch zu erklären, dass ausschließlich naturalistische Studien in die Metaanalyse eingegangenen sind und daher Aussagen über Behandlungseffekte nicht möglich waren.

Interessanterweise fand eine Metaanalyse von Ponce-Correa et al. [70] keinen signifikanten Zusammenhang zwischen verschiedenen Maßen für subjektive Recovery und Remission der Symptomatik, wohingegen Patienten mit einem höheren psychosozialen Recovery auch eine deutlichere subjektive Recovery berichteten. Dieser Befund deutet darauf hin, dass die Symptomreduktion, welche ja ein wesentliches Ziel der psychiatrisch-psychotherapeutischen Behandlung ist, möglicherweise wenig mit dem subjektiven Genesungsprozess der Patientinnen und Patienten oder deren Sicht auf die Genesung zu tun hat. Der aufgezeigten Beziehung zwischen psychosozialer Funktionalität und subjektiver Recovery könnte die Fähigkeit, Beziehungen zu anderen Menschen aufzubauen und aufrechtzuerhalten, als gemeinsamer Mechanismus zugrunde liegen (vgl. [55]). Des Weiteren könnten die beruflichen und beschäftigungsbezogenen Elemente, welche die Konzeptualisierung psychosozialer Funktionalität mitbestimmen, mit Aspekten der persönlichen Genesung wie Empowerment, Fähigkeit zur Entwicklung von Zielen und Lebenszielen jenseits der Behandlung konvergieren.

## Somatische Komorbidität

Weltweit sind Menschen mit einer Schizophreniespektrumsstörung von einer gegenüber der Allgemeinbevölkerung um ca. 15 bis 20 Jahre reduzierten Lebenserwartung [67] und einer erhöhten Belastung durch Behinderungen betroffen [38], welche im Zusammenhang mit einem erhöhten Risiko für körperliche Erkrankungen und einem eingeschränkteren Zugang zur Gesundheitsversorgung stehen [25, 26, 53]. Zusätzlich tragen eine stigmabedingte Unterdiagnostik/-behandlung und verminderte Selbstfürsorge zur erhöhten Mortalität bei. Die meisten Metaanalysen und Übersichtsarbeiten zur körperlichen Gesundheit von Menschen mit psychischen Erkrankungen konzentrieren sich auf kardiovaskuläre oder metabolische Erkrankungen in Ländern mit hohem Einkommen und zeigen deren hohe Prävalenz bei Patientinnen und Patienten mit Erkrankungen aus dem Schizophreniespektrum auf, welche das Risiko für eine somatische Begleiterkrankung erhöhen [14]. Zu den wichtigen externen Einflussfaktoren gehören Adipositas, ungesunde Ernährungsgewohnheiten, Rauchen, Bewegungsmangel und übermäßiger Alkoholkonsum [72].

Patientinnen und Patienten mit Erkrankungen aus dem Schizophreniespektrum weisen eine hohe Prävalenz von Adipositas und ungünstigen kardiometabolischen Parametern auf, welche das Risiko für eine somatische Begleiterkrankung erhöhen [14]. Zu den wichtigen externen Einflussfaktoren gehören ungesunde Ernährungsgewohnheiten, Rauchen, Bewegungsmangel und übermäßiger Alkoholkonsum [72]. Der langfristige Verlauf von Erkrankungen aus dem Schizophreniespektrum wird erheblich durch körperliche Begleiterkrankungen beeinflusst. Als Folge u. a. von Adipositas, Stoffwechselstörungen, Lungenerkrankungen und Herz-Kreislauf-Erkrankungen haben SSD(„schizophrenia spectrum disorders“)-Patientinnen und -patienten eine um etwa 15 bis 20 Jahre kürzere Lebenserwartung als die Allgemeinbevölkerung [67]. Zusätzlich tragen eine stigmabedingte Unterdiagnostik/-behandlung und verminderte Selbstfürsorge zur erhöhten Mortalität bei.

In der Literatur wird häufig ein Zusammenhang zwischen metabolischen Störungen mit der Verwendung von Antipsychotika hergestellt, da die wirksamsten Antipsychotika der zweiten Generation, wie Clozapin oder Olanzapin, zu Gewichtszunahme und Stoffwechselstörungen führen können [10, 54]. Allerdings zeigte sich in einer aktuellen Beobachtungsstudie von Taipale et al. [81], die eine große finnische Kohorte untersuchten, dass eine kontinuierliche antipsychotische Therapie das Risiko für körperliche Erkrankungen und die damit verbundene Sterblichkeit nicht erhöhen, sondern sogar deutlich reduzieren. Dennoch sollten die möglichen langfristigen Nebenwirkungen von Antipsychotika, insbesondere auf das Herz-Kreislauf-System, nicht vernachlässigt werden, da sie die Lebensqualität der Patientinnen und Patienten beeinflussen und einen wichtigen Aspekt im komplexen Gefüge von Faktoren, die den Langzeitverlauf mitbestimmen, darstellen. Daten der PAFIP-Studie legen nahe, dass insbesondere Gewichtszunahme im ersten Jahr einer antipsychotischen Therapie bedeutsam für den weiteren Verlauf sein könnte [83].

Die aktuellen Leitlinien zur Behandlung der Schizophrenie (z. B. Leitlinie der Deutsche Gesellschaft für Psychiatrie und Psychotherapie, Psychosomatik und Nervenheilkunde [16] oder des National Institute for Health and Clinical Exellence [66]) empfehlen Metformin als eine Option, um eine durch Antipsychotika verursachte Gewichtszunahme zu behandeln oder zu begrenzen. Kardiometabolische Risikofaktoren können v. a. mithilfe nichtpharmakologischer Interventionen beeinflusst werden, die beispielsweise Bewegung oder einen gesunden Lebensstil bei Personen und mit Erkrankungen aus dem Schizophreniespektrum fördern. Hierfür stehen psychotherapeutische Interventionen wie Psychoedukation, motivierende Gesprächsführung oder kognitiv-verhaltenstherapeutische Interventionen bezüglich Lebensstilveränderung zur Verfügung, mitunter auch in Kombination mit einer Förderung der körperlichen Aktivität. Bezüglich der Effekte solcher nichtpharmakologischen Interventionen, ihrer spezifischen Charakteristika und möglichen Langzeitergebnisse konnte eine aktuelle Metaanalyse einen generellen Nutzen in Bezug auf Maße wie Body-Mass-Index (BMI), Gewicht und Taillenumfang, die Schwere der psychotischen Symptome, die kognitive Leistung, körperliche Leistungsfähigkeit, das allgemeine Funktionsniveau und die Lebensqualität zeigen [20]. Dagegen ergaben sich keine Effekte in Bezug auf metabolische Parameter (außer Triglyceride), andere psychopathologische Symptome (z. B. Angst und Depression) und das Ausmaß der allgemeinen körperlichen Aktivität. Im Allgemeinen waren sowohl der direkte als auch der langfristige Nutzen größer bei Interventionen, die Bewegung (insbesondere moderates bzw. intensives aerobes Training) allein oder in Kombination mit Psychotherapie beinhalteten. Auch zeigte sich eine vergleichbare Wirksamkeit bei Personen mit einer ersten Krankheitsepisode und denjenigen mit chronischem Verlauf, ein Nutzen kann also in allen Stadien der Erkrankung und nicht nur in frühen Episoden erreicht werden.

## Neuropsychologie/kognitive Leistung

Probleme im Bereich der Neuropsychologie/Neurokognition sind eine sehr häufige zusätzliche Einschränkung bei Patientinnen und Patienten mit Störungen aus dem Formenkreis der Schizophrenien.

Die beschriebenen Defizite beeinträchtigen das soziale Funktionsniveau und die Alltagsbewältigung der Patientinnen und Patienten erheblich [65], wobei Defizite in der sozialen Kognition die Auswirkungen von Problemen in der Neurokognition auf das soziale Funktionsniveau noch verstärken (mediieren; [24]).

Epidemiologische längsschnittliche Kohortenstudien, die Kinder untersuchten, die später im Verlauf eine schizophrene Erkrankung entwickelten und mit Personen aus der Normalbevölkerung verglichen, konnten studienübergreifend bereits vor Beginn der Erkrankung Hinweise auf neurokognitive Defizite feststellen (narrative Übersichtsarbeit: [22]). Die frühesten Hinweise auf erste neuropsychologische Einschränkungen sind im Alter von 18 Monaten nachweisbar [60]. Insgesamt etwa die Hälfte der zu Erkrankungsbeginn messbaren neurokognitiven Defizite bestehen bereits im Kindesalter und verstärken sich besonders zwischen dem Auftreten erster prodromaler Symptome und der Manifestation einer ersten psychotischen Episode [22], während zwischen der ersten psychotischen Episode und späteren Episoden eine gewisse Stabilität im neuropsychologischen Verlauf besteht.

Längsschnittliche Studien über 10 bis 20 Jahre, die den Zeitraum von der frühen Kindheit bis zum mittleren Erwachsenenalter (30–50 Jahre) umfassen, weisen auf eine Verschlechterung des allgemeinen intellektuellen Funktionsniveaus und anderer neuropsychologischer Funktionsbereiche hin [23, 51, 56, 76, 87], wobei insbesondere performanzbasierte Fähigkeiten stärker und verbale kognitive Fähigkeiten weniger betroffen sind.

Im höheren Erwachsenenalter bestehen Hinweise darauf, dass sich neuropsychologische Funktionsbereiche bei Personen mit schizophrenen Störungen schneller abbauen als bei Personen aus der allgemeinen Normalbevölkerung [71, 78], allerdings ist die Anzahl der vorhandenen Studien sehr gering und es fällt schwer, altersgemäße neuropsychologische Probleme von dem mit der Erkrankung assoziierten Abbau abzugrenzen. Von einem beschleunigten neurokognitiven Abbau sind insbesondere Personen mit einer sehr langen Erkrankungsdauer betroffen, die häufig im sozialen Funktionsniveau stark eingeschränkt sind und/oder sozialpsychiatrisch betreut werden [27].

Im Bereich neurokognitiver Defizite besteht eine sehr starke Heterogenität zwischen verschiedenen Patientinnen und Patienten [35]. Fett et al. [22] entwickelten basierend auf Ergebnissen querschnittlichen Studien zur Leistungsheterogenität bei Personen mit schizophrenen Störungen vier neurokognitive Subgruppen:

- Personen, bei denen primär Gedächtnisprobleme bestehen,
- Personen mit primären Problemen im Bereich des Schlussfolgerns und Problemlösens,
- Personen mit globalen, schweren neuropsychologischen Defiziten (zwischen 9,9 und 40,2 %) und
- Personen ohne neuropsychologische Defizite (die etwa 30 % umfassen).

Basierend auf diesen Ergebnissen erscheint eine Differenzierung und Personalisierung neuropsychologischer Rehabilitationsangebote überaus sinnvoll, die krankheitsphasenspezifisch und möglicherweise auch subgruppenspezifisch angeboten werden könnten.

## Strukturelle Bildgebung andere biologische Variablen

Im Gegensatz zu klinischen und kognitiven Parametern liegen nur wenige Studien zum Verlauf hirnstruktureller oder -funktioneller Parameter vor, insbesondere zum prädiktiven Wert biologischer Variablen auf den Langzeitverlauf. Dies ist, etwa bei MR-Bildgebung, nicht zuletzt mit den technischen Einschränkungen bei Follow-ups verbunden.

Frühere Studien zum Verlauf der Schizophrenie in den ersten 5 Jahren legen präfrontale Volumenminderungen über den Follow-up-Zeitraum nahe, welche deutlich über die (altersbedingten) Veränderungen bei Kontrollpersonen hinausgingen [43]. Nachfolgende Studien zeigten ferner progressive Volumenreduktionen im Temporallappen, welche mit der Persistenz psychotischer Symptome (insbes. Halluzinationen) in Verbindung gebracht wurde [58]. In nachfolgenden größeren Studien (*n* > 200) konnten dann vor allem frontale und auch thalamische Volumenreduktionen der grauen Substanz, aber auch Reduktionen des Volumens der weißen Substanz in zahlreichen Hirnarealen nachgewiesen werden [58]. Zusätzlich konnte die Dauer erneuter psychotischer Episoden im Follow-up-Zeitraum mit frontalen Volumina korreliert werden [58]. Diese Befunde konnten auch über mehrere Arbeitsgruppen teilweise repliziert werden [69]. Einschränkend ist zu bedenken, dass zumindest zu einem Teil diese Veränderungen auch auf Medikationseffekte von Antipsychotika zurückzuführen sein könnten [28]. Jedoch zeigen Studien zu Risikopersonen und in der Frühphase der Erkrankung über kürzere Follow-Up-Intervalle, dass schon vor bzw. im Übergang zur Erkrankung morphometrische Veränderungen vorliegen, sodass diese nicht einfach nur Veränderungen aufgrund einer medikamentösen Intervention sind [5, 47, 52].

In neueren Studien liegen Analysen vor, welche einerseits die Follow-up-Zeiträume auf teils mehr als 10 Jahre ausdehnen, zum anderen auch in voxelweisen bzw. oberflächenbasierten Analysen eine genauere Lokalisation der Veränderungen erlauben. Aktuelle Befunde der norwegischen TOP-Studie, die von 79 Ersterkrankten mit einer psychotischen Störung und 218 Kontrollen jeweils 23 bzw. 77 Personen bis zu 10 Jahre mittels struktureller Magnetresonanztomographie (MRT) nachuntersuchen konnten, zeigen bei Erkrankten häufiger eine negative Abweichung von typischen Verlaufstrajektorien der kortikalen Dicke [4]. Insbesondere Personen mit schlechtem klinischem Verlauf zeigen eine (über bei Studieneinschluss vorliegende) weitere progressive Reduktion der Kortexdicke [73]. Ähnlich wie frühere Longitudinalstudien mittels MRT zeigen diese aber eine hohe Heterogenität der Veränderungen über mehrere kortikale Regionen hinweg [3] und eine Trennung konfundierender Effekte antipsychotischer und anderer Pharmakotherapien [46] schränkt die Aussagekraft weiterhin ein. Der Ansatz, biologische Variablen im Querschnitt bei unterschiedlichen Patientengruppen in unterschiedlichen Stadien zu betrachten, etwa für funktionelle MRT bei kognitiven Defiziten [86], erweist sich dabei nicht als Lösung des Problems fehlender Longitudinaldaten.

Auch genetische Ansätze wie polygene Risikoscores für Schizophrenie haben bisher weniger Varianzaufklärung zur Frage des Langzeitverlaufs nach psychotischer Ersterkrankung erbracht als klinische Variablen [12, 36, 75], wobei familiäre Häufung eine höhere Varianzaufklärung als polygene Scores zeigte [11, 12].

## Einfluss von Behandlung auf den Langzeitverlauf

Inwiefern die für die Akut- und Rezidivprophylaxe eingesetzten therapeutischen Ansätze auch tatsächlich eine Modifikation des langfristigen Krankheitsverlaufs über Jahrzehnte hinweg erreichen, ist ein zentrales Thema klinischer Forschung, positive Effekte sind aber derzeit nicht sicher nachgewiesen [32, 33, 59]. Einerseits zeigen Verlaufsstudien positive Effekte einer (medikamentösen) Langzeitbehandlung nicht nur auf das Risiko erneuter psychotischer Episoden, sondern auch auf die Mortalität der Betroffenen [62]; dennoch bleibt unklar, ob und wieviel die Therapiestrategien der letzten Jahrzehnte zur Verbesserung der Langzeitverläufe beigetragen haben.

Aus den aktuellen Longitudinaldaten, welche 10 oder mehr Jahre umfassen, ist jedoch ein Optimierungspotenzial für einen möglichen positiven Einfluss auf den Langzeitverlauf absehbar, bezogen auf die identifizierten Determinanten des Verlaufs. Hierzu gehört u. a. die Identifikation und individualisierte Behandlung etwa bei Therapieresistenz oder bei Untergruppen mit sich früh verschlechterndem Outcome. So konnte z. B. in einer Studie bei 102 Schizophreniespektrumspatientinnen und -patienten mit 10-Jahres-Follow-up gezeigt werden, dass trotz eines Anteils von 31 % als behandlungsresistent eingestufter Patientinnen und Patienten nur 7 % in diesem Zeitraum Clozapin erhalten hatten [85], obwohl dessen bessere antipsychotische Wirksamkeit in mehreren Studien nachgewiesen ist. Dies ist umso relevanter als sich Subgruppen mit früher vs. später Therapieresistenz abzeichnen [17]. Der Mangel an prospektiven Langzeitstudien ist dabei insbesondere für die viel diskutierte Frage nach der Modifikation von Krankheitstrajektorien ein Hindernis, jedoch nicht nur im Hinblick auf mangelnde (frühe) Therapieinterventionen, sondern auch die Frage, ob und welchen Subgruppen alternative (insbes. pharmakologische) Therapieschemata angeboten werden sollten bzw. ob und bei wem pharmakologische Erhaltungstherapien notwendig sind [84]. Unklar bleibt der Einfluss intermittierender antipsychotischer Therapie im Vergleich zu einer durchgehenden Medikation [29, 74] sowie der Dosisreduktion in der Rezidivprophylaxe [44] im Hinblick auf den Langzeitverlauf. Auch der Effekt gezielter Interventionen zur Reduktion nach Ersterkrankung, welche deutliche Vorteile für Betroffene zeigen, bedarf einer weiteren Evaluation zu Langzeiteffekten [7].

Auch für nichtpharmakologische Interventionen wie aerobes Training [18, 31], repetitive transkranielle Magnetstimulation ebenso wie besser etablierte und evidenzbasierte Ansätze wie die kognitive Verhaltenstherapie und Elektrokonvulsionstherapie, welche in der kurzfristigen Behandlung positive Effekte zeigen, liegen keine ausreichenden Studien zu Langzeiteffekten vor. Aktuelle randomisierte kontrollierte Pilotstudien unter Einbeziehung sozialpsychiatrischer Interventionen weisen auf günstigere Verläufe nach einem Jahr bei Ersterkrankten hin [68].

## Fazit

Der Langzeitverlauf der Schizophrenie(n) ist heterogen und verläuft keineswegs bei allen oder einer größeren Mehrheit von Betroffenen in chronifizierte oder behandlungsresistente Zustände. Vielmehr geben die aktuellen Longitudinalstudien nicht nur Anlass zur Hoffnung, dass mit gezielter und frühzeitiger Intervention nicht nur die Transition zur Erkrankung, sondern auch der weitere Langzeitverlauf aktiv beeinflusst werden können. Am Vielversprechendsten sind eine möglichst frühe Intervention mit Verkürzung des behandlungsfreien Intervalls bei beginnender psychotischer Episode, möglicherweise eine intensivierte Therapie in den ersten ein bis zwei Jahren nach Krankheitsbeginn und eine differenzierte und individualisierte (z. B. auf somatische Risikokonstellationen etc. ausgerichtete) Adaptation von Leitlinienprotokollen.

Aktuell erlaubt der Mangel an ausreichenden Längsschnittdaten über Jahrzehnte hinweg nur begrenzte Aussagen ob und wie deutlich die Spontantrajektorien wirklich modifiziert werden können. Die Verfügbarkeit neuer biologischer und statistischer Ansätze wird es in aktuellen Langzeitstudien erlauben, noch detaillierter deskriptive Verläufe und Prädiktoren über lange Zeiträume zu analysieren und den Fokus nicht nur auf eine kurzfristige Symptomreduktion, sondern mehr noch auf dauerhafte Erhaltung oder Erhöhung des psychosozialen Funktionsniveaus bzw. Recovery zu lenken. Schon jetzt kann aber in der Psychoedukation von Betroffenen und Angehörigen wie auch der Allgemeinbevölkerung noch mehr auf die Heterogenität der Outcomes und damit auf die Möglichkeit günstigerer Krankheitsverläufe bei einer nicht unerheblichen Zahl Erkrankter hingewiesen werden.
